# Prognosis vs Treatment Interaction

**DOI:** 10.1093/jncics/pky006

**Published:** 2018-04-28

**Authors:** Jack Cuzick

**Affiliations:** Centre for Cancer Prevention, Wolfson Institute of Preventive Medicine, Queen Mary University of London, Charterhouse Square, London, EC1M 6BQ, UK

## Abstract

There is a somewhat confused belief that a biomarker must show an interaction effect with a treatment before it can be used to determine the need for such a treatment. This is rarely true for well-established clinical markers such as tumor size or regional lymph node involvement. In many cases, this is also not true for biomarkers, especially when considering nontargeted therapies. Here I argue that for nontargeted treatments prognosis is often more important than interaction with treatment, because it is the absolute and not the relative benefit that matters, and when there is no treatment interaction, the same relative benefit translates into a larger absolute benefit for poor prognosis patients.

When determining the need for additional treatment, confusion exists between the importance of a marker capable of predicting the likelihood of disease recurrence (or progression) vs one that predicts an interaction with treatment response. The former is usually referred to as “prognostic value,” whereas the latter has often been referred to as “prediction” or, more fully, as “prediction of treatment response.” This is a misnomer that should be avoided. This is largely due to the fact that treatment effects need to be assessed in terms of absolute benefits, but most of the statistical models used are based on relative effect sizes. Thus a marker that is strongly prognostic for treatment failure or recurrence but has no interaction with treatment (ie, the relative effect sizes are similar for high- and low-risk patients) is still highly predictive of the absolute benefit of treatment. An example is node positivity in breast cancer. This is one of the strongest prognostic factors for recurrence and an important factor in deciding the need for chemotherapy, but there is no interaction with chemotherapy in the conventional multiplicative proportional hazards model. For example, in the Early Breast Cancer Trialists' Collaborative Group (EBCTCG) overview, the relative benefit of poly-chemotherapy was similar in node-positive vs node-negative women. For women younger than age 50 years, the hazard ratios for recurrence were 0.64 for N0 vs 0.63 for N+ women (1). For women age 50 to 69 years, there was a slightly and statistically nonsignificantly larger relative benefit in node-negative women (hazard ratio = 0.77 vs 0.83, *P* = .6), belying the fact that the absolute benefit was larger in the node-positive women (1.6% vs 0.8% per year, *P* = 1.4×10^-6^). Thus, while there is no interaction of nodal status with response to chemotherapy, it is clear that the absolute benefit of chemotherapy is much greater in node-positive women, because the same relative benefit translates into a larger absolute benefit. This is also true for mortality when comparing chemotherapies with or without anthracyclines and with or without taxanes (2). Similar results are also seen for tumor size and grade.

Similar principles are also applicable to a molecular biomarker or panel of biomarkers, but here the distinction between qualitative and quantitative biomarkers is important. A qualitative marker such as estrogen receptor or human epidermal growth factor receptor 2 (HER2) positivity indicates a qualitatively different type of the disease in which different pathways are activated for tumor growth. In the case where treatment attacks a specific pathway and the biomarker determines that that pathway is activated, the marker is truly predictive of response to a specific treatment (notably endocrine therapy for estrogen receptor–positive tumors and trastuzumab and related drugs for HER2-positive disease, and other monoclonal antibodies, eg, PD-1/PD-L1 blockade in melanoma) (3). However, more general quantitative markers, such as those measuring the rate of cell cycle proliferation or metastatic potential (which do not [yet] identify qualitatively different disease mechanisms) may not have any impact on the relative effect of a treatment in different subgroups, but by identifying a high-risk subgroup, they are still important in determining which patients will benefit most in absolute terms from additional treatment. This distinction has been more fully developed elsewhere (4–6), where the terms “therapy guiding” and “clinical utility” are used for markers that provide guidance on which patients might benefit most from treatment. Note that this does not require a significant interaction, but seeks to separate patients based on likely absolute benefits and/or harms.

For example, much has been made of the fact that the Oncotype test showed “predictive value for response to chemotherapy” in one trial when used alone (7). However, many newer tests have not demonstrated an interaction with treatment, but have shown similar prognostic value to Oncotype in the first five years of follow-up, and a greater ability to predict late recurrence (8,9). One of the problems is that the analysis of interactions with treatment depends heavily on the effect in the low-risk group, where there are few recurrences and substantial uncertainties in any conclusion. Another issue is that the interpretation of molecular tests always needs to be made in light of important standard clinical prognostic factors such as nodal status and tumor size, and in this trial the interaction between Oncotype and treatment was no longer significant after accounting for these factors (10).

Thus, quantitative markers that are strongly prognostic but do not demonstrate a statistical interaction with treatment can still be highly informative when making a decision about the use of chemotherapy. To base a decision only on a statistical interaction with treatment (often called predictive value) can be misleading.

## Notes

Affiliation of author: Centre for Cancer Prevention, Wolfson Institute of Preventive Medicine, Queen Mary University of London, Charterhouse Square, London, EC1M 6BQ, UK.

Prof. Cuzick’s institute has received support from Genomic Health, Myriad Genetics, and Nanostring to evaluate disease markers, and his institution receives a royalty from Myriad Genetics for development of a prognostic marker for prostate cancer, of which he receives a share.
